# Computerized analysis of hypomimia and hypokinetic dysarthria for improved diagnosis of Parkinson's disease

**DOI:** 10.1016/j.heliyon.2023.e21175

**Published:** 2023-10-23

**Authors:** Justyna Skibińska, Jiri Hosek

**Affiliations:** a Faculty of Electrical Engineering and Communication, Brno University of Technology, Technicka 12, Brno, 61600, Czechia; b Unit of Electrical Engineering, Tampere University, Kalevantie 4, Tampere, 33100, Finland

**Keywords:** Acoustic analysis, Facial analysis, Hypokinetic dysarthria, Hypomimia, Machine learning, Parkinson's disease

## Abstract

**Background and Objective:**

An aging society requires easy-to-use approaches for diagnosis and monitoring of neurodegenerative disorders, such as Parkinson's disease (PD), so that clinicians can effectively adjust a treatment policy and improve patients' quality of life. Current methods of PD diagnosis and monitoring usually require the patients to come to a hospital, where they undergo several neurological and neuropsychological examinations. These examinations are usually time-consuming, expensive, and performed just a few times per year. Hence, this study explores the possibility of fusing computerized analysis of hypomimia and hypokinetic dysarthria (two motor symptoms manifested in the majority of PD patients) with the goal of proposing a new methodology of PD diagnosis that could be easily integrated into mHealth systems.

**Methods:**

We enrolled 73 PD patients and 46 age- and gender-matched healthy controls, who performed several speech/voice tasks while recorded by a microphone and a camera. Acoustic signals were parametrized in the fields of phonation, articulation and prosody. Video recordings of a face were analyzed in terms of facial landmarks movement. Both modalities were consequently modeled by the XGBoost algorithm.

**Results:**

The acoustic analysis enabled diagnosis of PD with 77% balanced accuracy, while in the case of the facial analysis, we observed 81% balanced accuracy. The fusion of both modalities increased the balanced accuracy to 83% (88% sensitivity and 78% specificity). The most informative speech exercise in the multimodality system turned out to be a tongue twister. Additionally, we identified muscle movements that are characteristic of hypomimia.

**Conclusions:**

The introduced methodology, which is based on the myriad of speech exercises likewise audio and video modality, allows for the detection of PD with an accuracy of up to 83%. The speech exercise - tongue twisters occurred to be the most valuable from the clinical point of view. Additionally, the clinical interpretation of the created models is illustrated. The presented computer-supported methodology could serve as an extra tool for neurologists in PD detection and the proposed potential solution of mHealth will facilitate the patient's and doctor's life.

## 1 Introduction

Parkinson's disease (PD) is the second most frequent neurodegenerative disorder, with a prevalence of 2% for people aged over 65 years [1]. PD is associated with a progressive loss of dopaminergic neurons in substantia nigra pars compacta, which consequently causes cardinal motor symptoms such as bradykinesia, rigidity, resting tremor, or postural instability [2], [3], [4]. In addition to these symptoms, PD patients can experience other motor symptoms such as hypokinetic dysarthria, dysphagia, hypomimia, or PD dysgraphia [5], [6], [7], [8], [9]. With regard to the non-motor symptoms, PD patients may experience sleep disorders, cognitive deficits, hallucinations, constipation, and other issues [2].

Hypomimia is characterized by an expressionless face with little or no sense of animation [10]. In particular, it is linked to muscle stiffness, difficulties with facial movements, limited ability to raise eyebrows [11], problems with orofacial functions (e.g. movements of the jaw and lips [12] at a slower pace [13], [14], as well as jaw tremor [15]). The PD patients find difficulties in posed smiling and voluntary grinning [16]. Other typical symptoms include a lower blinking rate [17], [18], an unintentionally opened mouth, flattened nasolabial folds [19], and asymmetry in the face [20], [21]. The hypomimia could be also associated with challenges with emotional processing (inter alia subjective emotional experience (alexithymia) and facial recognition) [22].

Hypokinetic dysarthria (HD), the early symptom of PD [23], is a motor speech disorder that frequently accompanies hypomimia [5]. It is caused by a basal ganglia control circuit pathology [5] and occurs in up to 90% of PD patients [24]. It is manifested in the field of respiration, phonation, articulation, and prosody. More specifically, the following speech/voice disorders could be observed: airflow insufficiency, irregular pitch fluctuations, harsh and breathy voice quality, reduced loudness, monoloudness, monopitch, unnatural speech rate, improper pausing, and imprecise articulation. For a comprehensive review on HD we refer to [25], [26], [27], [28], [29].

Although curing PD patients is difficult, several treatment strategies (usually pharmacological or neurostimulation) have been proposed to improve the patient's quality of life [30]. To adjust the treatment policy and control its effect, patients visit a hospital several times per year. Nevertheless, this frequency is insufficient, and the patients under clinical examination could also be subjected to the Hawthorne effect [31]. In addition, patients could have more rapid neurodegeneration, severe motor fluctuations, and side effects (e.g., levodopa-induced dyskinesia (LID)) which all result in a detrimental impact on the patients' quality of life. Therefore, they should be quickly and effectively addressed. The LID commonly occurs among PD patients and is a type of dyskinesia associated with debilitating treatment by levodopa. It manifests mostly after long treatment. Nevertheless, it can show rarely after a few days or months of therapy. The most common symptom is choreiform movements [32]. The support system for levodopa change was presented in [33]. Moreover, to overcome the limitations of current strategies, researchers started to explore the benefits of mobile health (mHealth) applications in the remote monitoring of elderly and PD patients [34], [35], [36], [37]. While the acoustic analysis of HD in mHealth systems plays a significant role [38], [39], [40], [41]. Neither the remote assessment of hypomimia, nor the impact of the combination of both modalities has been inspected much. The audio signal was inter alia used to distinguish between PD, idiopathic rapid eye movement sleep behavior disorder (iRBD), and healthy control (HC) [42]. Smartwatches and mobile phones are suitable devices to differentiate between illnesses. Moreover, the fitness tracker and smartphone questionnaires were utilized to forecast the wearing-off period when the PD patient needs to take the next dose of levodopa. The researchers obtained average balanced accuracy of 70.0–71.7% for participant 1 and 76.1–76.9% for participant 2 [43]. Furthermore, Shimmer3 wearable device was used to collect data to predict the tremor severity level manifesting in PD. The over-sampling techniques and XGBoost allow authors for achieving 99% accuracy considering 16 patients [44]. Moreover, the actigraph device could be also used to detect PD based on sleep disorders [45]. Additionally, the smart insoles are applied to recognize PD based on gait analyzing. The authors in [44] used wavelet transforms and deep learning to distinguish between adult, elderly, and PD patients group considering gait abnormalities. The 29 records were analyzed. They succeeded with 96.5% accuracy for the distinction between classes [44].

To sum up, we identified the following knowledge gap: although HD and hypomimia are frequent symptoms of PD, to the best of our knowledge there are limited studies dealing with the combination of these modalities for the sake of improved diagnosis of PD [46]. Therefore our main goal is to explore the possibility of fusing computerized analysis of hypomimia and HD with the utility of a spectrum of various speech exercises, in order to propose a new methodology of PD diagnosis that could be easily integrated into mHealth systems (thanks to built-in microphones and cameras). We consider this as the first step towards a better remote assessment of PD symptoms. In addition, both modalities could be collected passively (e.g., during a video call) and with data anonymization (by applying parameterization), which further accents the advantages of this technology.

The contributions of this study are as follows:

1. The evaluation of a variety of state-of-the-art methods for PD detection based on hypomimia and hypokinetic dysarthria signs was illustrated.
2. The utility of the unique dataset is another advantage of this paper, which allows for identifying the most powerful speech exercise from a spectrum of exercises. We enrolled 46 HC and 73 PD patients. 43 speech exercises were evaluated.
3. The generated geometric features, here, facial landmarks, were computed with the respect to the anthropometry. The dynamics of changes of them were expressed by the calculated scalars.
4. In terms of audio features, the recommendations from [25] on how to extract parameters were applied to the unique speech exercises.
5. The simultaneous modeling of both modalities improved the accuracy of PD diagnosis with the usage of the XGBoost classifier.
6. The clinical interpretation of the digital biomarkers was presented with the usage of SHapley Additive exPlanations (SHAP) values and statistical analysis.
7. The tongue twister was indicated as the most significant speech exercise for PD detection, among the analyzed 43 tasks.
8. The proposed support methodology is a good starting point for the at-home monitoring PD system.

### 1.1 Computerized analysis of hypomimia

Hypomimia has been hitherto examined in the aspects of impairment of expressing emotion in PD patients. Most studies have examined differences in the capability of expressing emotions such as happiness, sadness, disgust, fear, anger, surprise, and neutrality. Although little work has been done in this area so far, some studies have been published (see Table 1). A state-of-the-art review of the computerized analysis of hypomimia is described in detail in [47].

**Table 1:** Summary of the papers related to analyzing hypomimia for PD patients.

Reference	No. of HC	No. of PD patients	Task	Access	Modality	Comment	Metrics
[50]	8	7	Expressing 6 emotions	Private	Video	Detection	Differences in group for the worked-out functions
[52]	17	17	Expressing emotions: anger, disgust, happiness, sadness	Private	Video	Detection	p-value <0.05
[53]	15	15	Watching funny movies and answering 5 questions	Private	Video	Detection	0.90-0.99 AUC
[57]	12	12	Posing with different emotional expressions	Private	Video	Detection	p-value <0.05
[19]	23	11	Selfie photos	Private	Image	Detection	p <0.05, 0.79 specificity/0.82 sensitivity, 0.58/0.54 sensitivity/specificity
[60]	50	50	Photo	Private	Image	Detection	67% accuracy
[61]	15	-	Watching cartoon	Private	Image	Detection	p-value <0.05
[50]	8	7	Watching movie clip	Private	EMG	Detection	p-value <0.05
[54]	543	61	Expressing 3 emotions	Private	Video	Detection	95.6%
[55]	39	47	Expressing neutral mimic and emotion	On request	Video	Detection	0.9991 F1-score, 0.9997 F1-score
[59]	0	727	Describing patients' negative or positive experience	Private	Video	Regression	0.560 Mean Absolute Error (MAE)
[62]	0	772	Describing patients' negative or positive experience	Private	Video, Audio	**Table:** Regression, Multiclassification	0.48 MAE, 0.55 F1
[63]	27	27	Assessment of hypomimia and influence of the medication on this symptom	Public/Private	Video	**Table:** Detection	for class detection: 0.75 AUROC, for comparison of the differences in state between medications: 76% off, 67% on
[56]	39	47	Neutral mimic and smiling	On request	Video	Detection	99.39% accuracy, 99.49 F1-score
[51]	75	91	Assessment of hypomimia and indication on valuable features	On request	Video	**Table:** Detection	0.87 AUC, 78.3% accuracy, sensitivity 79.1%, 77.8% specificity
[46]	**Table:** 112 ‘on’, 74 ‘off’	**Table:** 111, 74	Assessment of video and audio records	Private	Video, Audio	**Table:** Detection	**Table:** 0.85 AUROC for ‘on’ state, 0.90 AUROC for ‘off’ state
[64]	13	13	Assessment of eye fixation and gait patterns	Private	Video	**Table:** Detection	Accuracy, sensitivity and specificity up to 100%
[22]	17	40	Correlation of reduced facial expressiveness vs. altered emotion processing	Private	Video	Correlation analysis	p-value <0.05
[16]	16	15	Assessment of posed smiling and abnormalities of voluntary movements of the lower face	Private	3D-optoelectronic system, Infrared Video	Correlation analysis	p-value <0.05

Good candidates for the automatic assessment of hypomimia are the facial feature extraction methods that can be divided into two primary groups: 1) geometry-based and 2) statistics-based. The geometry-based methods typically use facial landmarks and then compute distances between those landmarks or measure areas between a couple of landmarks detected on the face. These distances can reflect anthropomorphic distances occurring on the human face [48]. The statistics-based group uses measurements based on changes in illumination between pixels [49].

There are currently multiple approaches for facial emotion analysis, including facial electromyography (fEMG), affectograms, facial action coding system (FACS), automatic maximally discriminative facial movement coding systems (MAX), or automatic facial expression recognition (FER) with the use of machine learning (ML) techniques. The image and video-based approaches can be divided into two types: 1) methods using detection of points of interest in a face, followed by ML modeling; and 2) deep convolutional neural networks (CNNs) that learn feature extraction directly from the image data [47].

Available works dealing with the assessment of hypomimia based on the analysis of emotions use relatively simple methods. In [50], the authors used a function of frequency, duration, and intensity of FACS as the measure of facial expressivity to distinguish between HC and PD patients. In total, 6 emotions were studied, including amusement, sadness, anger, disgust, surprise, and fear. The introduced method seems to allow differentiation between HC and PD patients based on quantitative analysis. 7 PD patients and 8 HC just took part in the experiment. The task of the participants was the self-evaluation of emotions after watching the movie clips. This is a subjective part of the conducted test.

The work in [51] created 12 markers based on 68 facial landmarks of two types: distances and areas. In the research, there were involved 91 de-novo and drug-naive patients and 79 HC with the limitation to people suffering from depression. The record of the Czech native speaker contains a one-minute monologue. The binary logistic regression was applied as a classifier, with the leave-one-subject-out cross-validation, and with 5 features. They obtained 0.87 Area Under the Curve (AUC). The accuracy was equal to 78.3%, sensitivity was 79.1% and specificity was 77.8%. The dynamic of the face was not expressed by the proposed features. Additionally, the type of cross-validation, leave-one-subject-out, could slightly overfit the results.

The authors of [52] studied the variation from the normal state and state when expressing emotions for the PD patients and HC. 17 cases per group were examined for this study. To evaluate changes in facial expressivity, the Euclidean distance between a neutral state and the expression of given emotions was computed with the created features vectors. The differences between HC and PD patients (for both acting and imitating emotions) were regarded as significantly different according to the two-tailed t-test. This study also found that the most impaired emotions in PD patients are anger and disgust.

Another study also used Action Units (AUs) [53] and subsequently applied ML techniques for the creation of a support decision system. The data were gathered with a three dimensional (3D) sensor and linear regression was used as a classifier. The detection was relatively accurate - it reached AUC between 0.90 and 0.99. However, in this case, the size of the experiment was 15 PD patients and 15 HC. This methodology is dependent on particular hardware (3D sensor) and proprietary software.

Another approach, which used FACS and ML, was presented in [54]. This time the data were gathered by the webpage tool.^1^ The gathered dataset contained 1812 videos for 604 participants: 61 PD patients and 543 HC. The record of one patient contains a 10–12 s video, which presented a video of a smiling, disgusted and surprised face. The emotion was repeated 3 times with a break for the neutral face. The authors measured the variation in the facial muscle movements, however, by grading the expression of the emotions with the AU (0-5). The research proved, thanks to the interpretability of logistic regression, that three AU during smiling brings valuable information for this exercise for detecting PD. It was AU_01, AU_06, AU_12, and AU_04 for disgusted. The paper also included classification tasks with the Support Vector Machines (SVM), nevertheless after balancing the dataset on the whole data thanks to the Synthetic Minority Oversampling Technique (SMOTE) algorithm. The leave-one-out cross-validation brought 95.6% accuracy for this approach.

The next solution with the combination of geometric and texture features was proposed in [55]. The dataset contains samples from 39 HC and 47 PD patients. The difference between neutral and expressed emotion was measured by the facial expression factors (FEFs) and facial expression change factors (FECFs), respectively – for geometric features. For texture features, an extended histogram of oriented gradients (HOG) was computed. It was based on three dimensions: HOG-XY, HOG-YT, and HOG-XT. The authors used Principal Component Analysis (PCA) and ML classifiers as well as 5-fold cross-validation. The outcome of classification was the best for the fusion of texture and geometric features for Random Forest (RF) 0.9991 F1-score and SVM 0.9997 F1-score. Notwithstanding, the authors mentioned that unfortunately the PCA performed unclearly, with the possibility of overfitting.

The extended study from [55], which is including end-to-end learning, is presented in [56]. The authors proposed the Semantic Feature based Hypomimia Recognition Network (SFHR-NET). Belonging to this architecture is, inter alia the Semantic Feature Classifier (SF-C), to adjust the feature-salient map. Additionally, they used Progressive Confidence Strategy (PCS) to balance the semantic loss and classification loss. Further, the neural network (NN) contains RGB spatial representation (spatial encoder) and optical flow (temporal encoder). Moreover, the interpretability of the approximate activated area was possible by Gradient-weighted Class Activation Mapping (GRAD-CAM). For the experiment, the authors used 39 HC and 47 PD patients. The training test contained 60% data, validation 10%, and testing 30%. The mentioned end-to-end solution contains Visual Geometry Group (VGG) as the backbone, segmenter, SF-C, PCS, and optical flow, giving 99.39% accuracy and 99.49% F1-score. However, the cross-validation was not performed.

The authors of [57] measured entropy from video records of 12 PD patients and 12 HC, and they examined their faces during smiling. This procedure allowed them to examine the reduction of facial movement thanks to the measurement of changes in pixel intensity. This paper concluded that bradykinesia and reduced facial movement (entropy) was registered for PD more frequently in comparison to HC for all of the studied emotions (i.e., happiness, sadness, fear, anger, disgust, surprise [57]).

Another alternative to the video analysis approaches is an electromyography (EMG) based experiment. Its analyses were published in recent years in [50], [58]. The authors measure a difference in the activity of the facial muscles. This method is, unfortunately, less comfortable for patients than the previously mentioned methods. Participants in the experiment were asked to report on their emotional states throughout the experiment. The proposed methodology is relatively subjective without defining a unified speech exercise.

All the previous methods dealt with PD detection. However, recently there were also some studies that tried to measure the progress of PD [59]. One example of such work is presented in [59]. In this study, a relatively significant number of patients was used (727). Only PD patients were included, and HCs were missing. In the experiment, subjects had to describe their negative or positive experience by themselves. Researchers used video and measured facial features such as the width or height of the mouth, eyebrow, or eye for each video frame. For the analysis ML regression was used, in particular, the Random Forest Regressor.

Another study [62] was involved in PD progress evaluation, which tried to classify PD patients into four classes based on the progress of the disease. This was based on facial expressivity ratings. This dataset included 772 records of 117 PD patients. These subjects conducted an exercise where they talked about their positive or negative memories. This approach was multimodal, where the authors were combining audio and video data. The reported F1-score for multiclassification was equal to 0.55. For regression of the progress, they used the Hierarchical Bayesian neural network (HBNN-C) [62].

In another paper [63], the transfer learning methodology was used to detect PD. The authors trained CNN on the database YouTube Faces Database, which contains images extracted from 3425 videos of 1595 people. The VGG Neural Network was trained on them. The videos from YouTube were collected to gather PD patient's records, 107 in total. The density distribution of the predicted score of hypomimia was produced as the result. The network was tested on the 27 PD patients and 27 HC. The classification with labels provided by two neurologists was equal to 0.75 area under the receiver operating characteristic (AUROC). However, the dataset was not unified in the clinical understanding. The Tufts Clinical data were used for evaluation of the effect of medication in PD, with a mean of 3639 frames per video in a clinical interview. In the cohort of 33 patients (The Tufts Clinical Data), 76% of the cases were detected in those off medication and 67% in those on medication [63]. This tool could serve as the measure of the influence of treatment on PD patients.

The multimodal approach of PD detection based on video and audio modalities was introduced in [46]. The training dataset consisted of the records of 112 PD patients during the ‘on’ phase and 111 HC. Additionally, 74 records of PD patients during the ‘off’ phase and 74 HC were gathered for the validation dataset. The video recordings of reading the text by the participants were captured by the smartphone. 20 features were extracted for classification purpose. To them belong inter alia: age, gender, reading time, pause percentage, average pitch, pitch variance, phonetic score, voice volume variance, 6 key eye- and mouth-related features. The nine classifiers were utilized with 10-fold cross-validation. For the training dataset, the distinction of the PD patients from HC was possible with the 0.85 AUROC thanks to the Logistic Regression. Whereas, the differentiation between the PD patients and HC for the validation dataset was achieved with 0.90 AUROC thanks to the AdaBoost classifier.

Moreover, the paper [64] presented the another multimodal approach for PD detection. The authors utilized the records of gait and eye fixation. In the study, 13 PD patients and 13 HC participated. The authors used the ocular fixation evaluation in the research, which is the ability to keep the stare at the concrete point. The microsaccades eye movement has typically frequency of the movement 1-2 Hz for HC, whereas for the PD patients are recognized intervals of 5.7 Hz [64], [65]. The two types of features were extracted: deep features computed from the convolutional neural network, and kinematic features calculated from optical flow. Next, covariance matrices were computed based on the spatial distribution of these features. Subsequently, the temporal mean of covariance matrices is calculated as the final features. The Random Forest was chosen as a classifier together with cross-validation leave-one-patient-out. The accuracy of PD detection reached up to 100%.

Table 1 gives a summary of the research papers mentioned previously above about hypomimia in PD. The table also illustrates the fact that access to PD patients is mostly limited in the research and this same collection of related data.

### 1.2 Computerized analysis of hypokinetic dysarthria

Other promising digital biomarkers of PD are based on speech/voice analysis. Recently, the members of the Speech and Movement Disorders Study Group published guidelines for acoustic analyses in dysarthrias (including HD) of movement disorders [66]. The guidelines recommended several basic acoustic measures such as the mean intensity, standard deviation of intensity, standard deviation of fundamental frequency, jitter, shimmer, harmonic-to-noise ratio, diadochokinetic rate, diadochokinetic regularity, vowel space area, voice onset time, among others, that quantify HD in the field of phonation, respiration, articulation and prosody.

Moreover, the power of oscillation in the range of 2-6 Hz was examined for the sustained phonation task – emission of vowel ‘e’ in [67]. The higher value of this parameter occurs to be characteristic of essential tremor (ET). 58 patients with ET and 74 HC were taken under analysis. Furthermore, the existing differences were checked for patients under and not under treatment. The classifier SVM together with Correlation Features Selection and 10-fold cross-validation were used to distinguish the classes. The achieved accuracy between the group achieved more than 80.0%, 89.5% sensitivity, and 74.2% specificity.

Furthermore, the influence of medication on PD mid-advanced patients was evaluated in [68]. Additionally, the distinction between early-stage PD patients and HC was carried out. 115 Italian PD patients and 108 Italian HC were included in the study. The symptom HD was the foundation of the research. The vowel and sentence were the utilized speech exercises. The authors extracted 6139 features. The results for SVM and 10-fold cross-validation were as follows:the differentiation between early-stage PD achieved 81.5% for vowel and sentence, the diversification between mid-stage PD and HC obtained 93.5% for vowel and 81.5% for sentence, whereas the distinction between mid-stage PD ON vs. OFF medication reached 92.6% for vowel, 72.4% for sentence.

In addition, the impact of medication was analyzed in [69]. The speakers were Italian and 266 HC and 160 PD took part in the experiment. The participants performed the pronunciation of the vowel ‘e’ for 5 s. 453 various speech features were extracted, and among them, the most important occurred to be mel-frequency cepstral coefficients (MFCC), fundamental frequency (F0), shimmer, jitter, wavelet decomposition measures, low-frequency tremor, and glottal-to-noise excitation (GNE). The 10-fold cross-validation together with traditional machine learning classifiers achieved for SVM and the distinction between early PD vs. HC 0.83% accuracy. The differentiation between midstage PD patients ON medication vs. OFF was possible at 0.79 accuracy for k-nearest neighbors algorithm (KNN). The obtained results were registered for standard ML algorithms than the combination of mel-spectrograms and CNN.

A decision-support system based on the basic features was proposed in [39]. The authors used a smartphone to assess speech/voice in 30 HC, 30 PD patients, and 50 subjects with iRBD. The group with iRBD was included because it is one of the early markers of PD. The sustained phonation of vowel [a], diadochokinetic exercise – repetition of pa-ta-ka, and monologue were used as the tasks. The results of classification between PD and HC were 75.0% sensitivity, 78.6% specificity, and 0.85 AUC with the usage of logistic regression. The authors proved the advantages of using smartphone technology for prodromal diagnosis of PD with an observation that biomarkers as the monopitch, decreased rate of follow-up intervals and inappropriate silences are the most valuable. Additionally, the authors reported discrimination between PD and iRBD subjects with 66.7% sensitivity, 71.0% specificity, and 0.78 AUC.

Besides the above-mentioned parameters, researchers usually extend the feature set by additional measures or introduce completely new ones. For instance, a new set of clinically interpretable articulatory kinetic biomarkers was introduced in [70]. They were extracted from diadochokinetic speech exercise in a cohort of 50 PD patients and 50 HC. Two main dependencies were evaluated. First, the velocity of the mid-term air pressure was explored in the light of the possibility of evaluating the kinetics of the speech of PD patients. For this purpose, the envelope of the speech was used because of its relationship with the mid-term airflow pressure. Secondly, the envelope of the speech was regarded as indirectly connected to the distributions of forces controlling the articulators, which vary between PD patients and HC. The extracted kinetic biomarkers were fed into SVM classifiers with a linear kernel while employing the sequential floating feature selection. The proposed features enabled the identification of PD with 85% accuracy.

In [71] the authors introduced new features based on the spectro-temporal sparsity characterization. The parametric sparsity measures (the shape parameter of a Chi distribution or the shape parameter of a Weibull distribution) and non-parametric sparsity measures (Gini-index, l1-norm, Shannon entropy) were calculated in the light of the fact that the speech spectral coefficients in PD are less temporally sparse than in HC. The dataset used for this purpose contained 45 HC and 45 PD patients. The participants were Colombian Spanish native speakers [72]. With the usage of SVM with a radial basis kernel function, the authors obtained 83.3% classification accuracy. The most informative features were the Gini index and parametric sparsity measures (shape parameters of the Chi and Weibull distribution).

Another feature, in the automatic diagnosis of PD, in this case based on the biomechanical model of speech and articulation, was utilized in [73]. Based on the relationship between formant oscillations and jaw-tongue reference position displacements, the authors introduced the absolute kinematic velocity (AKV) and reported (in a cohort containing 16 PD patients and 16 age- and gender-matched HC) that this measure has better discrimination properties than conventionally used vowel space area or formant centralization ratio.

With the increasing popularity of deep learning, some of the recent studies dealing with the automatic diagnosis of PD from speech/voice explore the utilization of deep neural networks (DNN). In [74], the authors studied the ability of CNN to model articulatory impairments. For this purpose, they used a multilingual dataset containing 50 PD and 50 HC Colombian, 88 PD and 88 HC German, and 20 PD and 15 HC Czech subjects. The CNN was fed by short-time Fourier transform (STFT) and wavelet transform representations of transitions between the onset and offset of phonation. With this approach, the authors achieved up to 89% classification accuracy. The same team later reported that the accuracies could be increased by up to 8% when employing transfer learning [75].

Another unique approach was introduced by Moro-Velazques et al. [76], who used the forced Gaussian based methodology to compare independently different phonetic units between PD and HC. In a multilingual corpus containing 47 PD and 32 HC Spanish, 50 PD and 50 HC Colombian, and 20 de-novo PD and 14 HC they reached up to 87% AUC when considering cross-corpora validation.

The research which took into consideration the gender shows that women suffering from PD characterize high-frequency content of speech, whereas low frequency content is typical for men patients [29], [77]. The authors of this study analyzed the four datasets and consider confounding factors. Moreover, the study in [78] proved that women with PD have better vocal control. 60 male and 40 female PD patients and this same HC individuals were taken under the analysis.

Generally, the field of computerized diagnosis of PD from speech/voice experiences is increasing in interest. The above-mentioned studies were just examples of some recently published works. For a comprehensive review, we refer to the following papers [25], [27], [28].

## 2 Methods

**Figure 1:**
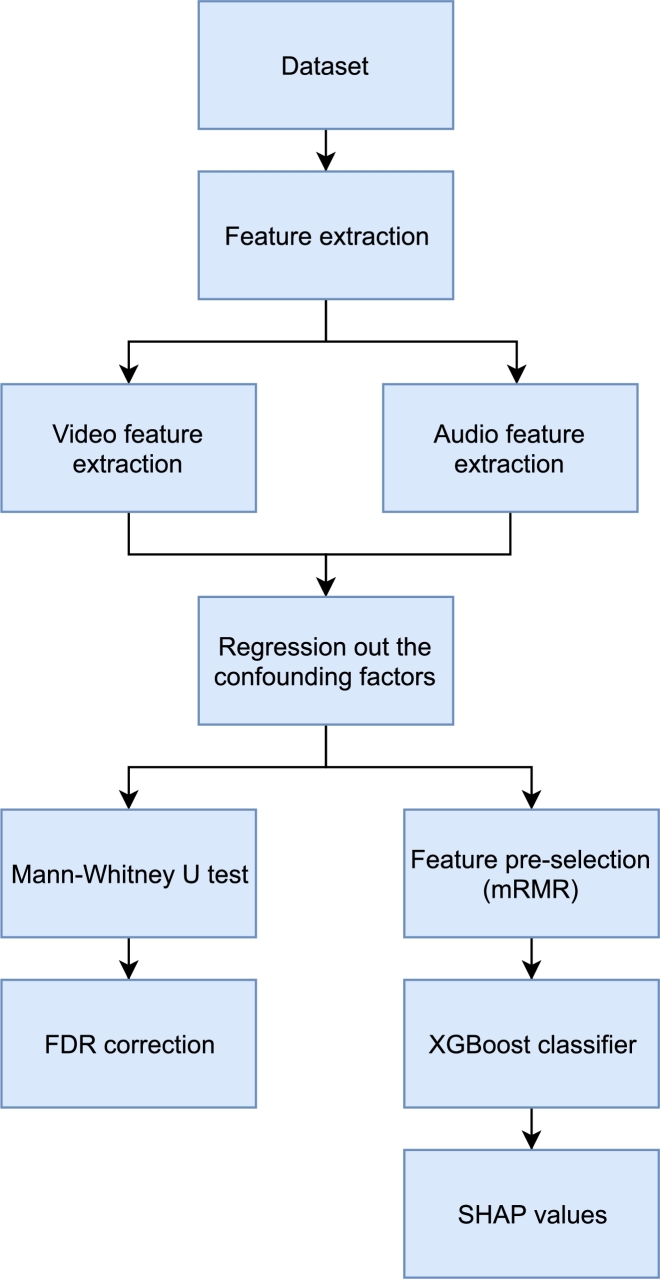
Flow of the algorithm.

The objective of this work was to create a methodology for PD detection using a multimodal combination of audio and video. For this purpose, we created a dataset, which includes PD patients and HC. We proposed 43 speech exercises and evaluated them using ML and statistics. This section describes the above-mentioned parts in detail and is structured as follows. The next subsection, 2.1, describes a dataset, how it was created and how it was split into training and validation parts. The next subsection, 2.2, contains feature extraction for video and audio modality and 2.3 describes the ML approaches used, the optimization techniques used, and a statistical evaluation of the models. A scheme of the conducted experiment is shown in Fig. 1.

### 2.1 Datasets

**Figure 2:**
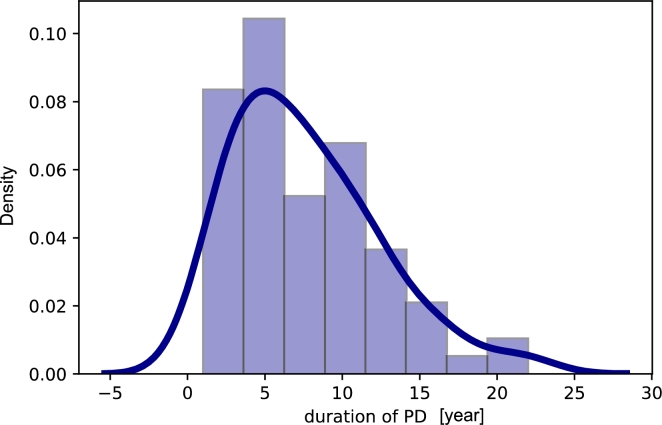
Kernel Density Estimation of duration of PD.

**Figure 3:**
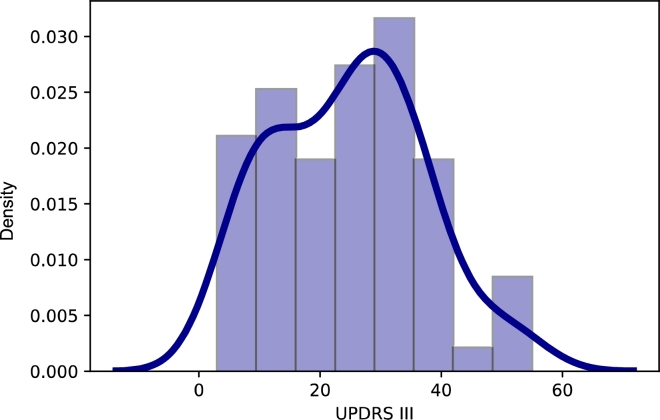
Kernel Density Estimation of level of UPDRS.

The data were gathered by the physicians in the Department of Neurology, Hospital in Czechia. The dataset had been started collected in 2009. The used scale of PD evaluation is the Unified Parkinson's Disease Rating Scale (UPDRS) [79], when the new, released in 2007, Movement Disorder Society-Sponsored Revision of the Unified Parkinson's Disease Rating Scale (MDS-UPDRS) was still relatively unfamiliar [80]. UPDRS is in the form of a questionnaire and could be found in [79]. The collected dataset contained records from the camera and microphone. Video and audio recordings were obtained for 43 speech exercises. We enrolled 46 HC (22 females [mean age 62 ± 9.02, range 42] and 24 males [mean age 66 ± 9.17, range 34]) and 73 PD patients (30 females [mean age 68 ± 8.20, range 37; education length 13.04 ± 2.70, range 9] and 43 males [mean age 66 ± 7.83, range 41; education length 14.76 ± 2.97, range 9]). A detailed description of the demographic and clinical data of the enrolled participants can be found in Table 2. The kernel density estimation of the duration of PD and level on the UPDRS III, are shown in Figure 2, Figure 3, respectively.

**Table 2:** Statistical and demographic description of the PD data.

	Mean	Std	Min	Q1	Median	Q3	Max	Range
Age	66.90	7.95	49	62.00	67.0	72.00	82	33
Duration of PD	7.80	4.39	1	4.00	7.0	11.00	22	21
UPDRS III	24.91	11.91	3	14.75	25.5	33.00	55	52
UPDRS IV	3.16	2.73	0	0.00	3.0	5.00	10	10
FOG	7.16	5.79	0	2.00	7.0	11.00	20	20
NMSS	38.37	23.06	2	19.00	34.5	54.00	112	110
RBDSQ	3.79	3.21	0	1.00	3.0	6.00	13	13
LED [mg]	1006.04	542.94	0	621.25	879.5	1325.50	2275	2275
ACE-R	87.15	8.01	60	82.75	87.5	93.00	100	40
MMSE	28.04	2.38	16	28.00	29.0	29.00	30	14
BDI	10.41	6.06	0	6.00	9.0	13.50	27	27
DX	74.32	8.90	30	71.00	76.0	79.00	88	58

The mean duration of PD is 7.80 years, and the mean UPDRS III [81] is 24.91, whereas UPDRS IV is equal to 3.16 (Table 2). The mean freezing of gait (FOG) [82] is equal to 7.16. The mean Non-Motor Symptoms Scale (NMSS) [83] is 38.37, and the mean REM sleep behavior disorder screening questionnaire (RBDSQ) [84] is equal to 3.79. The mean Levodopa Equivalent Dose (LED) for PD patients is equal to 1006.04 mg. The mean Addenbrooke's Cognitive Examination-Revised (ACE-R) [85] is equal to 87.15. The mean Mini-Mental State Examination (MMSE) [86] is equal to 28.04. The mean Beck Depression Inventory (BDI) [87] is 10.41. The mean Dysarthria Index (DX)[88] is equal to 74.32.

All of the participants in the experiment were involved in various speech exercises, and a wide range of different experiments was examined, including vowels, words, sentences, tongue twisters, and textual readings, as well as poems, free speech, diadochokinesis tasks, and others. The language used was Czech, but as we show later in this paper, the acoustical performance of the exercise is more important than its meaning. The details of the conducted speech exercises are presented in Table 4, Table 5. Video recordings were acquired using PANASONIC SDR-H20 with the sampling frequency of 25 frames per second (FPS). Audio recordings were gathered separately using a cardioid microphone (M-AUDIO Nova) placed on the arm within a distance of 20 cm from the patient's mouth, with a sampling frequency of 48 kHz and a 16-bit resolution. A trained acoustic engineer parametrized the signals using Praat [89] and Matlab functions [90], without viewing the patient's clinical data. The gathering of the data had ethical approval from the Ethics Committee of Masaryk University. Moreover, written consent has been obtained from all participants.

### 2.2 Feature extraction

To quantify PD, we designed several approaches for the extraction of features from audio and video. These were finally represented as the tabular values suitable for training ML models. Details for video extraction are presented in subsection 2.2.1 and for audio extraction in subsection 2.2.2.

#### 2.2.1 Facial features

**Figure 4:**
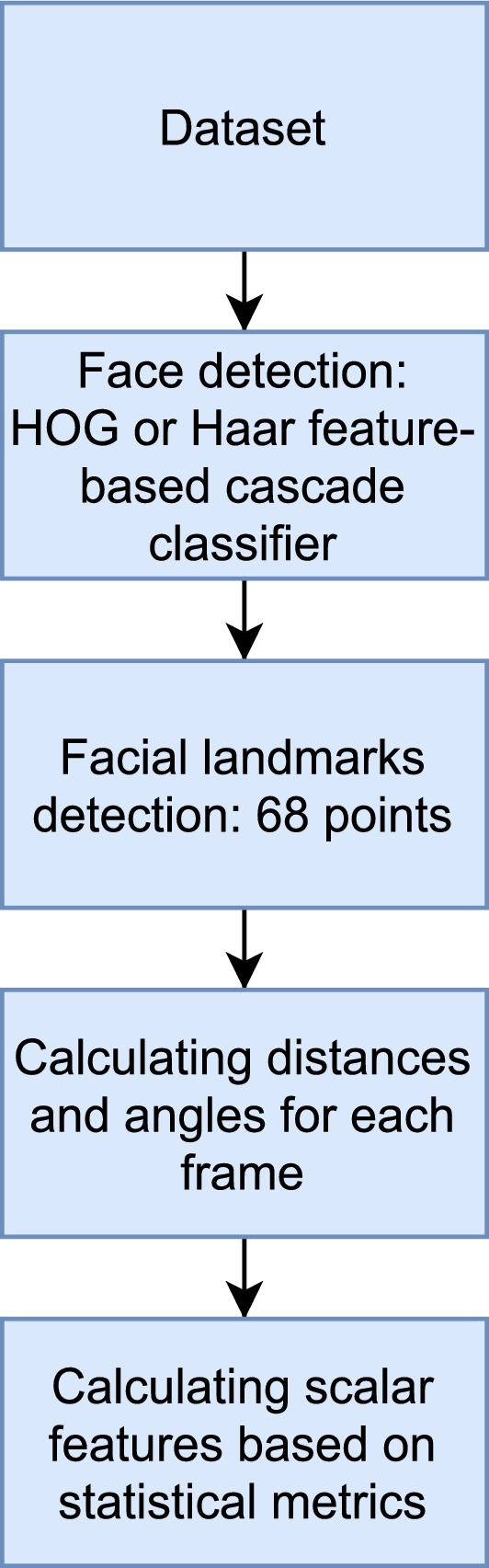
Flow of the facial features extraction.

**Figure 5:**
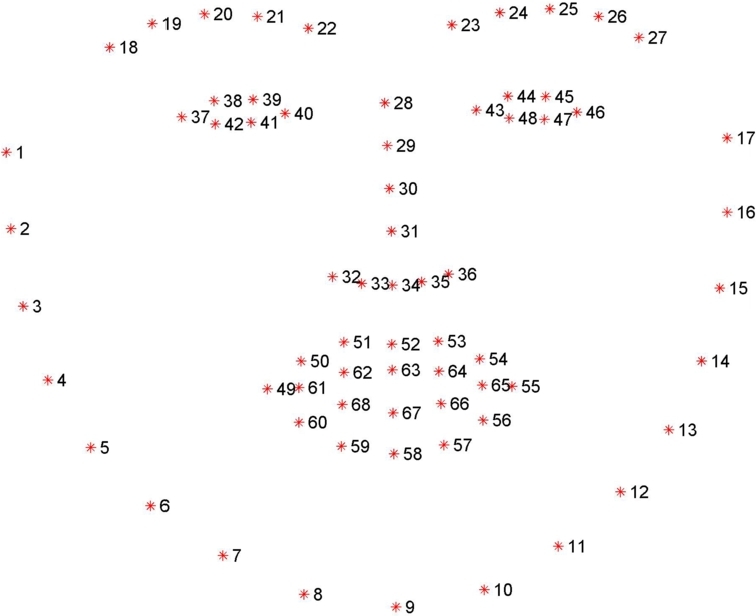
Facial features - illustration [108].

Our approach for video focused on facial features extraction, which was based on detecting characteristic points of the face. For the reproducibility of the experiment, we chose a open-source software,^2^ which located 68 points on the face. The scheme and placement of the points are presented in Fig. 5. The process of locating facial landmarks can be divided into two parts: facial detection and recognition of facial landmarks. For facial detection, we chose the HOG and Haar feature-based cascade classifiers. To extract (x,y) coordinates of facial features, facial landmark detectors were used. In particular, we used a neural network. To extract facial landmarks from each frame (i.e., we created a time series based on the video). Lighting was not considered. Nevertheless, the facial landmarks were detected anyway. The next step computed the distances, angles, and areas based on the 68 extracted facial landmarks (see Table 3). To compensate for the movement of the head, the distances were divided by the face length. Then, we analyzed and evaluated the future trained model. For this purpose, the following values were computed for each time-series: mean, standard deviation (std), relative standard deviation (rsd), minimum (min), maximum (max), range, variance (var), the slope of the function in time, as well as Shannon entropy (se) [91], [92] and approximate entropy (ae) [93], [94], [95], which are considered valuable for short medical time-series analysis [96] (see Fig. 4).

**Table 3:** Explanation of the extracted features.

Name feature	Points, angle
D1	37, 49
D2	46, 55
D3	22, 23
D4	52, 58
D5	20, 38
D6	25, 45
D7	39, 41
D8	45, 47
D9	31, 9
D10	1, 17
D11	18, 22
D12	23, 27
D13	34, 52
EYEBROW1	Angle: (22, 19) vs. (40, 43)
EYEBROW2	Angle: (22, 19) vs. (23, 26)
EYEBROW3	Angle: (22, 19) vs. (23, 26)
EYEBROW4	Vertical: 19, 37
EYEBROW5	Vertical: 26, 46
EYE1	37, 38
EYE2	37, 39
EYE3	46, 45
EYE4	46, 44
EYE5	40, 39
EYE6	40, 38
EYE7	43, 44
EYE8	43, 45
EYE9	37, 42
EYE10	37, 41
EYE11	43, 48
EYE12	43, 47
EYE13	40, 41
EYE14	40, 42
EYE15	46, 48
EYE16	46, 47
EYE17	38, 42
EYE18	45, 47
EYE19	39, 41
EYE20	44, 48
EYE21	37, 40
EYE22	43, 46
M1	49, 52
M2	49, 58
M3	55, 52
M4	55, 58
M5	49, 55
M6	52, 58
M7	60, 54
M8	50, 56
RATIO_MOUTH	M5/M6
MOUTH_AREA	The area of the inside of the mouth
LEYE_AREA	The area of the left eye
REYE_AREA	The area of the right eye
RATIO_FACE	D1/D2
RATIO_MOUTH _SKEW_UP	M3/M1
RATIO_MOUTH _SKEW_DOWN	M4/M2

#### 2.2.2 Voice features

The acoustic features were chosen according to a recommendation from [25]. All of them were computed from stored audio records in the dataset. Among those HD dimensions, i.e., articulation, prosody, phonation, parameters responsible for individual impairments were calculated, namely: airflow insufficiency, aperiodicity, inappropriate silences, increased noise, irregular alternating motion rate, irregular pitch fluctuations, microperturbations in amplitude, microperturbations in frequency, monoloudness, monopitch, the rigidity of tongue and jaw, slow alternating motion rate, tremor of the jaw and unnatural speech rate. The details could be found in Table 6, Table 7. In particular, the codes of acoustic features for them together with the information for which speech disorders are typical are presented in Table 6. Whereas, the descriptions of which acoustic features are specific for vocal tasks likewise speech disorders could be found in Table 7.

### 2.3 Statistical analysis and machine learning

For a better understanding of this subsection, the processing steps have been previously shown in Fig. 1.

PD depends on many factors, including age and gender. For this reason, it was necessary to eliminate the influence of these factors on the gathered data. Therefore, the confounding effect was removed from the audio and video. We used the regression out method for this purpose [97], [98]. The removal of the confounding effect eliminates the influence of confounding variables (age and gender) on the independent variables (extracted features) and dependent variables (the occurrence of PD). This method eliminates the need to create sex- and age-matches datasets. The linear regression model was fitted on each feature with confound as a predictor. The details of the method can be found in [99], [100].

The next step was to check the correlation of single features on PD. For this purpose we used the Mann-Whitney U test, a non-parametric test [101]. Thanks to this test, it was possible to check whether the two distributions for this same feature were statistically different. Additionally, the false discovery rate (FDR) correction was used to reduce the influence of rejecting a true null hypothesis. In the next step, features were preselected using the maximum relevance minimum redundancy (mRMR) algorithm [102]. Fifty of the most relevant features were chosen. Next, we used a machine-learning algorithm (in particular, the XGBoost classifier), as well as the Stratified 10-fold Cross-Validation for statistical evaluation. The stratified sampling (equal distribution of the representants of each class in the training and test dataset) and standardization of the data were applied during performed cross-validation [103]. The XGBoost is a classifier, that uses a kind of end-to-end tree ensembling model and has several advantages, such as the ability to capture non-linear dependencies in the data, the ability to deal with an imbalanced dataset, and the ability to offer efficient and robust solutions for structured data [104]. To interpret of the model, a game theory-based framework was computed, the so-called SHAP values [105], [106], which allow users to gather information on the correlations between the most important features relative to the model, positive or negative.

### 2.4 Metrics

The objective of this paper was to accurately classify between PD and HC subjects. The metrics for evaluation of the model were as follows: accuracy (Eq. (1)), sensitivity (Eq. (2)), specificity (Eq. (3)), and metrics dedicated to an imbalanced dataset: balanced accuracy (Eq. (4)) and the Matthews correlation coefficient (MCC) (Eq. (5)). Balanced accuracy was used as one of the hyperparameters during the training of the model for boosting the prediction.

Accuracy:

(1) $$Accuracy=\frac{TP+TN}{TP+TN+FP+FN}$$

Sensitivity:

(2) $$Sensitivity=\frac{TP}{TP+FN}$$

Specificity:

(3) $$Specificity=\frac{TN}{TN+FP}$$

Balanced Accuracy:

(4) $$BA=\frac{Sensitivity+Specificity}{2}$$

The MCC:

(5) $$\text{MCC}=\frac{\text{TP}⁎\text{TN}-\text{FP}⁎\text{FN}}{\sqrt{\left(\text{TP}+\text{FP})(\text{TP}+\text{FN})(\text{TN}+\text{FP})(\text{TN}+\text{FN}\right)}}$$

## 3 Results

This section is divided into two parts. First, analysis of the data using the Mann–Whitney U test is shown, which validates whether the features are statistically different between PD patients and HC. Then results achieved with different ML algorithms are presented. Clinical importance of the results and their interpretation are discussed later in section 4.

The results of the univariate analysis are presented in Table 8. We analyzed more than 200 different audio features and 550 different video features for each speech exercise. The Mann–Whitney U test was performed on the whole set of features and exercises after the regression out (i.e., it is the reason why some results are of negative value).

For the audio section, we selected the top 10 most statistically important features. Nine of the 10 features passed the test according to the p-value with FDR correction, with the threshold for the accepted significance level set to α=0.05. Table 8 also shows results of p-value and compares the results achieved using p-value with FDR correction. As explained in section 2.3, p-value measure with FDR correction is preferred for feature selection. Shimmer (TSK15) and relF0SD (TSK7) stand for phonation tasks, and we computed for the relative std of the fundamental frequency and the amplitude perturbation quotient, respectively. The description and broader definition of the features is provided in Table 4, Table 6.

**Table 4:** Carried-out vocal tasks.

Code	Vocal task	Description
TSK1	expiration	maximum phonation of [m] in one breath
TSK2	expiration	maximum phonation of [i] in one breath
TSK3	phonation	vowel [a] (sustained and normal intensity)
TSK4	phonation	vowel [e] (sustained and normal intensity)
TSK5	phonation	vowel [i] (sustained and normal intensity)
TSK6	phonation	vowel [o] (sustained and normal intensity)
TSK7	phonation	vowel [u] (sustained and normal intensity)
TSK8	phonation	vowel [a] (sustained and maximum intensity)
TSK9	phonation	vowel [e] (sustained and maximum intensity)
TSK10	phonation	vowel [i] (sustained and maximum intensity)
TSK11	phonation	vowel [o] (sustained and maximum intensity)
TSK12	phonation	vowel [u] (sustained and maximum intensity)
TSK13	phonation	vowel [a] (sustained and minimum intensity, but not whispering)
TSK14	phonation	vowel [e] (sustained and minimum intensity, but not whispering)
TSK15	phonation	vowel [i] (sustained and minimum intensity, but not whispering)
TSK16	phonation	vowel [o] (sustained and minimum intensity, but not whispering)
TSK17	phonation	vowel [u] (sustained and minimum intensity, but not whispering)
TSK18	diadochokinesis (DDK)	DDK pa-ta-ka
TSK19	rhytmical units	read poem
TSK20	main intonation pattern	same sentence read as interrogative
TSK21	main intonation pattern	same sentence read as imperative
TSK22	main intonation pattern	same sentence read as declarative
TSK23	intonation variability	monitoring prosody (declarative read sentence)
TSK24	intonation variability	monitoring prosody (imperative read sentence)
TSK25	intonation variability	monitoring prosody (imperative read sentence)
TSK26	intonation variability	monitoring prosody (interrogative read sentence)
TSK27	intelligibility of repeated words	repeated word complicated for the articulation
TSK28	intelligibility of repeated words	repeated word complicated for the articulation
TSK29	intelligibility of repeated words	repeated word complicated for the articulation
TSK30	intelligibility of repeated words	repeated word complicated for the articulation
TSK31	intelligibility of repeated words	repeated word complicated for the articulation
TSK32	intelligibility of repeated words	repeated word complicated for the articulation
TSK33	intelligibility of repeated words	repeated word complicated for the articulation
TSK34	intelligibility of repeated words	repeated word complicated for the articulation
TSK35	intelligibility of repeated words	repeated word complicated for the articulation
TSK36	intelligibility of repeated words	repeated word complicated for the articulation
TSK37	intelligibility of repeated sentences	repeated sentence complicated for articulation
TSK38	intelligibility of repeated sentences	repeated sentence complicated for articulation
TSK39	intelligibility of repeated sentences	repeated sentence complicated for articulation
TSK40	intelligibility of repeated sentences	repeated sentence complicated for articulation
TSK41	intelligibility of repeated sentences	repeated sentence complicated for articulation
TSK42	monitoring intelligibility and articulation	long read paragraph
TSK43	**Table:** interview at the beginning - monitoring prosody, hesitations, time needed for response, etc.	**Table:** free speech, usually the answer to “What are your hobbies?”, “Where do you come from?”, etc.

We approached the video content in a similar way. From the video features, we again selected 10 of the most important. The most valuable for this test was rsdD8 (TSK31), which stood for the task of the intelligibility of repeated words, and we computed a rsd in changes in the height of the eyelid. The p-value with FDR correction for α=0.05 was 0.0733 for video; however, the tests were passed for the p-value without FDR correction for the best ten features. The best of these are presented in Table 8. Additionally, the median value and interquartile range (IQR) are depicted separately in the table for HC and PD subjects.

To statistically evaluate achieved results, we applied the XGBoost algorithm. The training was done using stratified 10-fold cross-validation. The advantage of the XGBoost algorithm is that it uses specific loss function as function approximation as well as regularization techniques [109]. This same algorithm has special potential to perform the best among classical ML algorithms. Moreover, the SHAP values could clarify the decision standing behind the classification made by the algorithm. The possible use of the deep neural network would be limited by the size of the dataset and interpretation of the outcome usable for clinicians.

The models were evaluated separately for speech, for video, and also for a multimodal approach. The results are shown in Table 9. As is obvious from the table, the best-balanced accuracy was achieved with the multimodal approach. For balanced accuracy, we achieved (0.83 [0.11]), sensitivity (0.88 [0.13]), specificity (0.78 [0.20]) and MCC (0.68 [0.22]). The lowest value in the sense of balanced accuracy was registered for speech modality, i.e., (0.77 [0.11]).

**Figure 6:**
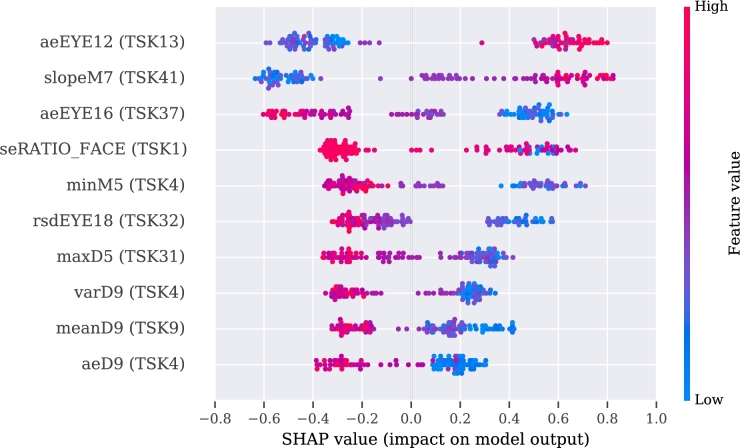
SHAP's values for the best 10 features from the video modality.

**Figure 7:**
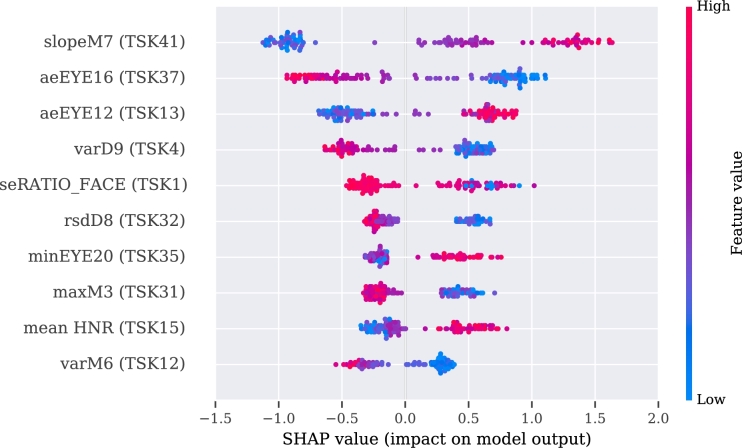
SHAP's values for the best 10 features from the multimodality.

**Figure 8:**
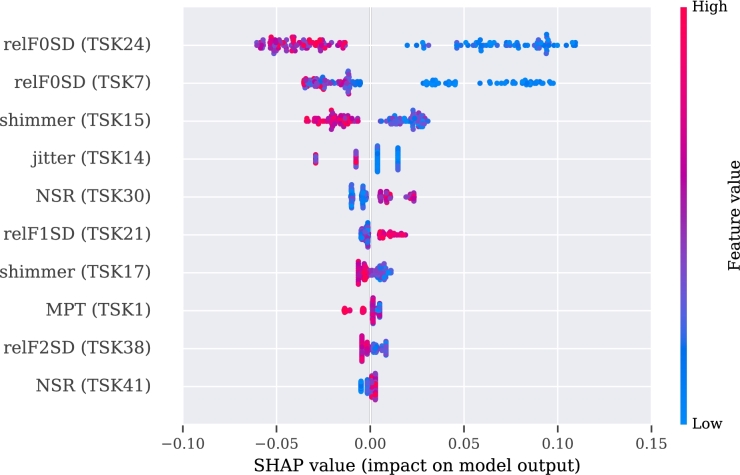
SHAP's values for the best 10 features from the audio approach.

To make these three models more interpretable, we used SHAP values for their analysis. The explanation of the video, audio, and multimodal models are presented in Figure 6, Figure 8, Figure 7, respectively.

The description of the SHAP values is provided for each modality separately, with an indication when two or more features correlate positively or negatively with PD. If the value of the feature is high and is registered on the positive side of the SHAP values, then PD is regarded as positively correlated with the feature [106].

For the video model, the approximate entropy of the change in distance of the (right or left) eyelid during the pronunciation of vowel ‘a’ (aeEYE12 (TSK13), see Table 3, Table 4) and the slope of the function of the skew distance of the mouth during pronunciation of a tongue twister (slopeM7 (TSK41), see Table 4, Table 5) indicate a positive correlation with the disease. The sentence was, in our case, in the Czech language, but more important than its meaning is its acoustic information, and difficulty in facial expression during pronunciation. The pronunciation of the exercise can be heard at the Google translate website.^3^ A negative correlation with PD was observed for the approximate entropy of changes of the eyelid during pronunciation of another Czech sentence (aeEYE16 (TSK37), see Table 3, Table 5). Furthermore, for the speech model, a positive correlation was registered for the net speech rate of a word, which indicates the intelligibility of repeated words (NSR (TSK30), see Table 6, Table 4, Table 5) and relative std of the 1st formant for the main intonation pattern (relF1SD (TSK21), see Table 6, Table 4). A negative correlation was registered for relative std of fundamental frequency for the intonation variability task (relF0SD (TSK24), see Table 6, Table 5) and relative std of fundamental frequency for pronunciation of the vowel ‘u’ (relF0SD (TSK7), see Table 6, Table 4).

**Table 5:** Meaning of the part of the exercises in Czech and English.

Code	In Czech	English translation
TSK19	**Table:** Chcete vidět velký lov? Budu lovit v džungli slov. Osedlám si Pegasa, chytím báseň do lasa.	**Table:** Would you like to see a big hunt? I will be hunting in a jungle of words. I will saddle the Pegasus, I will catch a poem into a lasso.
TSK20	Prostřete k obědu?	Will you lay the table?
TSK21	Prostřete k obědu!	Lay the table!
TSK22	Prostřete k obědu.	Lay the table.
TSK23	Teď musíš být chvíli trpělivý, než to dokončíme.	Now you have to be patient for a while until we finish.
TSK24	Tak dáš mi už konečně pokoj!	I urge you to leave me alone.
TSK25	Už mě to nebaví, dej mi už konečně pokoj!	I am fed up, I urge you to leave me alone.
TSK26	Tak co, jak to dopadlo?	So, what happened?
TSK27	rychlonožka	lightfoot
TSK28	marnotratný	wasteful
TSK29	horolezectví	mountaineering
TSK30	stříbrotepec	silversmith
TSK31	železobetonový	iron-concrete
TSK32	zákonodárce	legislator
TSK33	horkovzdušný	convection
TSK34	strastiplná	tortuous
TSK35	záviděníhodný	enviable
TSK36	československý	Czechoslovak
TSK37	Do čtvrt hodiny tam byla smršť.	In a quarter of an hour there was a whirlwind.
TSK38	Prohovořte to s ním dopodrobna.	Discuss it with him in detail.
TSK39	Při ústupu pluku duní bubny.	Drums are pounding during the retreat of regiment.
TSK40	Kuchařští učni nejsou jak zlatničtí.	Apprentices of cookery school are not as those from goldsmith one.
TSK41	Celý večer se učí sčítat.	He is learning to add the whole evening.

**Table 6:** Description of acoustic features. Details of features implementation are provided in [107].

Code of Acoustic feature	Description of the features	HD dimension	Specific disorder
DDK rate	DDK rate	articulation	slow alternating motion rate
DDK reg	std of DDK cycle periods	articulation	irregular alternating motion rate
DUV	fraction of locally unvoiced frames	phonation	aperiodicity
MPT	total speech time	phonation	airflow insufficiency
NSR	net speech rate	prosody	unnatural speech rate
SPIR	speech index of rhytmicity	prosody	inappropriate silences
jitter	period perturbation quotient	phonation	microperturbations in frequency
mean HNR	mean of harmonic-to-noise ratio	phonation	increased noise
relF0SD	relative std of fundamental frequency	prosody	monopitch
relF1SD	relative std of 1st formant	articulation	rigidity of tongue and jaw
relF2SD	relative std of 2nd formant	articulation	rigidity of tongue and jaw
relSEOSD	relative std of short-time energy	prosody	monoloudness
shimmer	amplitude perturbation quotient	phonation	microperturbations in amplitude

**Table 7:** Definition of the acoustic features in detailed.

HD dimension and specific disorder	Vocal tasks	Acoustic feature	Feature definition
**Phonation**			
Airflow insufficiency	Expiration with closed (TSK2) or opened (TSK3) lips	MPT	Maximum phonation time, aerodynamic efficiency of the vocal tract measured as the maximum duration of the sustained vowel/consonant.
Irregular pitch fluctuations	Sustained phonation (TSK3 - TSK17)	relF0SD	The standard deviation of fundamental frequency relative to its mean, variation in frequency of vocal fold vibration.
Microperturbations in frequency	Sustained phonation (TSK3 - TSK17)	jitter	Frequency perturbation, the extent of variation of the voice range. Jitter is defined as the variability of the F0 of speech from one cycle to the next. In this case it is implemented as the five-point period perturbation quotient.
Microperturbations in amplitude	Sustained phonation (TSK3 - TSK17)	shimmer	Amplitude perturbation, representing rough speech. Shimmer is defined as the sequence of maximum extent of the signal amplitude within each vocal cycle. In this case implemented as the five-point amplitude perturbation quotient.
Tremor of jaw	Sustained phonation (TSK3 - TSK17)	relF1SD, relF2SD	The standard deviation of the first (F1) and second (F2) formant relative to their mean. Formants are related to the resonances of the oro-naso-pharyngeal tract and are modified by position of tongue and jaw.
Increased noise	Sustained phonation (TSK3 - TSK17)	mean HNR	Harmonics-to-noise ratio, the amount of noise in the speech signal, mainly due to incomplete vocal fold closure. HNR is defined as the amplitude of noise relative to tonal components in speech.
Aperiodicity	Sustained phonation (TSK3 - TSK17)	DUV	Degree of unvoiced segments, the fraction of pitch frames marked as unvoiced.
**Articulation**			
Rigidity of tongue and jaw	Rhytmical units (TSK19), Basic intonation template (TSK20 - TSK22), Reading with different emotions (TSK23 - TSK26), Repeated word complicated for articulation (TSK27 - TSK36), Repeated sentence complicated for articulation (TSK37 - TSK41), Reading paragraph (TSK42), Monologue (TSK43)	relF1SD, relF2SD	The standard deviation of the first (F1) and second (F2) formant relative to their mean. Formants are related to the resonances of the oro-naso-pharyngeal tract and are modified by position of tongue and jaw.
Slow alternating motion rate	Diadochokinetic task (TSK18)	DDK rate	Diadochokinetic rate, representing the number of syllable vocalizations per second.
Irregular alternating motion rate	Diadochokinetic task (TSK18)	DDK reg	Diadochokinetic regularity, defined as the standard deviation of distances between following syllables nuclei.
**Prosody**			
Monoloudness	Rhytmical units (TSK19), Basic intonation template (TSK20 - TSK22), Reading with different emotions (TSK23 - TSK26), Repeated word complicated for articulation (TSK27 - TSK36), Repeated sentence complicated for articulation (TSK37 - TSK41), Reading paragraph (TSK42), Monologue (TSK43)	relSEOSD	Speech loudness variation, defined as the standard deviation of intensity contour relative to its mean.
Monopitch	Rhytmical units (TSK19), Basic intonation template (TSK20 - TSK22), Reading with different emotions (TSK23 - TSK26), Repeated word complicated for articulation (TSK27 - TSK36), Repeated sentence complicated for articulation (TSK37 - TSK41), Reading paragraph (TSK42), Monologue (TSK43)	relF0SD	Pitch variation, defined as the standard deviation of F0 contour relative to its mean.
Inappropriate silences	Basic intonation template (TSK20 - TSK22), Reading with different emotions (TSK23 - TSK26), Repeated sentence complicated for articulation (TSK37 - TSK41), Reading paragraph (TSK42)	SPIR	Number of speech inter-pauses per minute.
Unnatural speech rate	Basic intonation template (TSK20 - TSK22), Reading with different emotions (TSK23 - TSK26), Repeated word complicated for articulation (TSK27 - TSK36), Repeated sentence complicated for articulation (TSK37 - TSK41)	NSR	If we consider net speech time (NST) as a duration of speech without pauses, then the net speech rate (NSR) is defined as the number of phones per NST.

According to the results obtained from the multimodal approach, a positive correlation with PD subjects was identified for the slope of the function of the skew distance of the mouth during pronunciation of a tongue twister (slopeM7 (TSK41), see Table 3, Table 5) and the approximate entropy of the changes in the distance of the (right or left) eyelid during pronunciation of vowel ‘a’ (aeEYE12 (TSK13).

A negative correlation was recognized in the approximate entropy of changes of the eyelid during pronunciation of another Czech sentence (aeEYE16 (TSK37), see Table 3, Table 5) as well as for the variance in the distance between the nose and the end of the jaw during pronunciation of vowel ‘e’ (varD9 (TSK4), see Table 3, Table 4). Additionally, also visible among the ten best features is the influence of the audio features on the SHAP values, i.e., positive correlation of the mean of the harmonic-to-noise ratio during pronunciation of vowel ‘i’ (mean HNR (TSK15), see Table 4, Table 6).

Results of PD detection and its statistical evaluation are presented in Table 10, Table 11, Table 12. The experiment covers each vocal task and they were evaluated separately for each modality (audio and video) and for all the modalities together. The results obtained thanks to video modality are presented in Table 10; results obtained audio modality are shown in Table 11, and results using the multimodal approach (i.e., audio & video) are shown in Table 12.

The best result with video modality was achieved according to balanced accuracy with the task TSK39. It was a tongue twister (language of the exercise was Czech, see Table 5). For sensitivity, we achieved the best results with TSK4 (i.e., ‘a’ vowel, see Table 4) at 0.81 (0.14). For specificity, TSK1 (see Table 4) performed best at 0.69 (0.21), and for MCC, task TSK39 (see Table 5) performed best at 0.47 (0.29). The rest of the results for video are presented in Table 10. The top 10 most accurate speech exercises are described below in the text.

Table 11 includes the outcomes of models based on the audio dataset. The best results were the following: balanced accuracy 0.68 (0.13), specificity 0.66 (0.22), and MCC 0.36 (0.26), which were achieved for TSK7 (pronunciation of vowel ‘u’, see Table 4, whereas sensitivity was equal to 0.77 (0.13) for TSK24 (Czech sentence, see Table 5).

The results for this kind of approach for multimodality are presented in Table 12. Another Czech tongue twister found to be the most successful for such a multimodal approach is TSK41, see Table 5). This tongue twister even outperformed another speech task. The achieved balanced accuracy was 0.74 (0.13) and MCC 0.49 (0.27). Sensitivity was the best for TSK23 0.83 (0.14), which is a task for monitoring prosody thanks to intonation variability (see Table 4). Specificity was the best for TSK22 0.69 (0.22) which is a Czech sentence and indicates on an intonation pattern (see Table 4, Table 5). For better visualization of the results, the ten best speech exercises according to balanced accuracy are presented in Table 13. In this case, the multimodal approach was used. A better result was obtained for 5 cases out of 10 results. For the rest, there was no decrease in balanced accuracy, which remained approximately at the same level.

**Figure 9:**
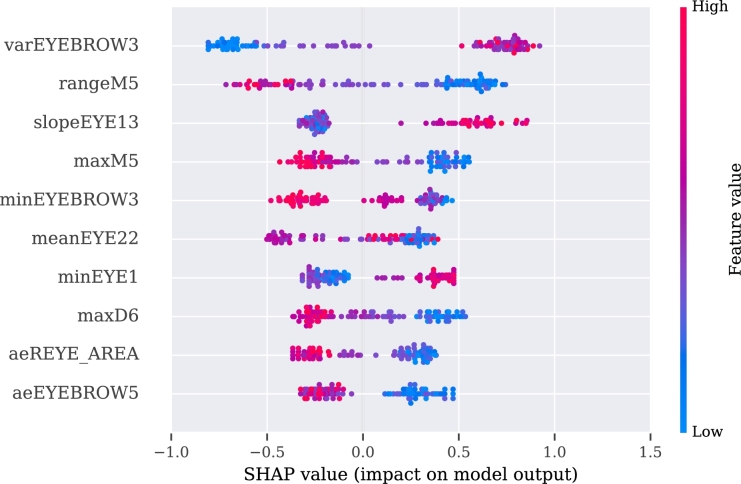
SHAP's values for the best video approach (TSK39).

**Figure 10:**
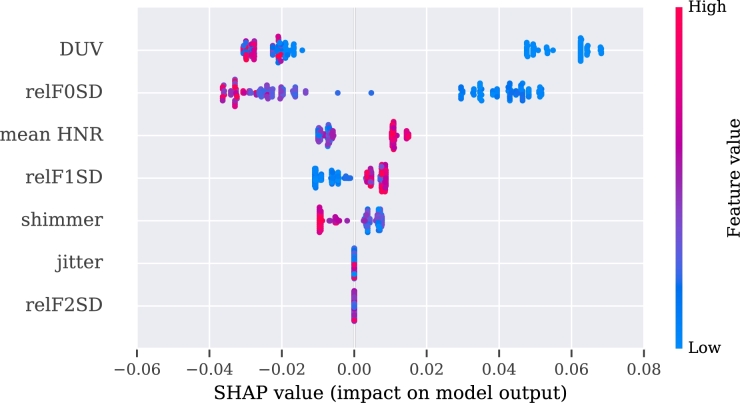
SHAP's values for the best audio approach (TSK7).

**Figure 11:**
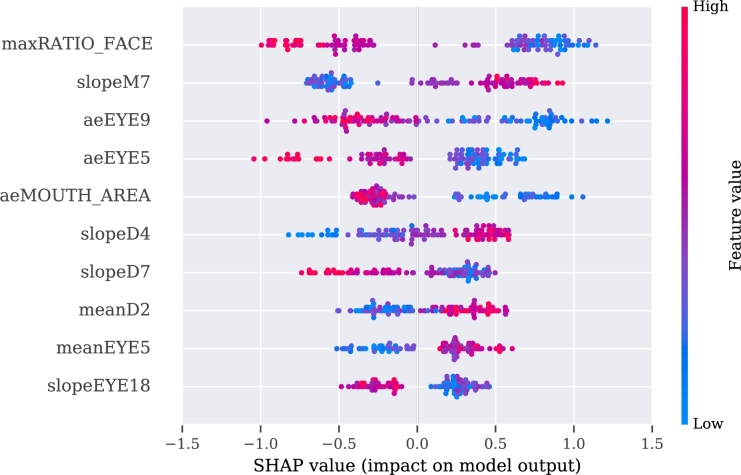
SHAP's values for the best multimodal approach (TSK41).

The SHAP values for the most predictive speech exercises for each modality are presented in Fig. 9 for video, Fig. 10 for audio and Fig. 11 for multimodality.

From the point of view of the video modality, the most significant speech exercise was identified as the difficult-to-pronounce sentence TSK39 (see Table 5, Table 4). The most positively correlated features with PD in this case were the variance in the angle between two of the eyebrows (varEYEBROW3, see Table 3) and the slope of the function of the changes in the moving of the eyelid (slopeEYE13, see Table 3). Negatively correlated is the range (rangeM5) and maximum (maxM5) of the width of the mouth.

For the audio model trained on separate speech exercises, the most valuable was the pronunciation of vowel ‘u’ (TSK7). Mean of harmonic-to-noise ratio (mean HNR) and relative std of first formant (relF1SD) were found to be positively correlated, whereas the fraction of locally unvoiced frames (DUV) and the relative std of the fundamental frequency were found to be as negatively correlated (relF0SD).

The best predictive speech exercise for the multimodal approach was identified as TSK41. Again, it is a difficult-to-pronounce sentence (see Table 4, Table 5). Positively correlated were the slopes of the functions of the changes in the skew distance of the mouth (slopeM7) and in the height of the mouth (slopeD4). Negatively correlated were the maximum in the ratio between the distances between the outer corner of the mouth and eye for the same side (maxRATIO_FACE) as well as the approximate entropy of the changes in eyelid EYE9 (aeEYE9).

## 4 Discussion

Thanks to testing various combinations of the speech exercises and selected features, a model that provides the optimal results for this dataset has been achieved. The introduced support methodology is a good inception to at-home monitoring PD patients. The dataset which was researched is unique and contains a bunch of Czech 43 speech exercises. The 46 HC and 73 PD were included in the study. The created geometric features maintain the anthropometrical character. The differences in the dynamic of facial expressions were evaluated thanks to the computed scalars. Regarding the audio features, the prompts from [25] were implemented how to generate valuable parameters. The utility of the multimodal approach together with the XGBoost classifier allows for outperforming the methodology based on a single modality. The SHAP values likewise statistical analysis provided the interpretability of the biomarkers. Moreover, the difficult-to-pronounce speech exercise – tongue twister occurred as the most beneficial speech task. Furthermore, the best-balanced accuracy was achieved using a multimodal approach, which was trained on the data extracted from the merged set of features. Based on the Mann–Whitney U test, we concluded that occurrence of shimmer (i.e., amplitude perturbation quotient), is a valuable feature for the distinction between PD and HC in the case of audio analysis. According to the p-value results with FDR correction for the nine features mentioned in the previous section, the statistical difference between the distribution of HC and PD patients is visible. Whereas, meaningful features were found for the pronunciation of vowels, tongue twisters (in the Czech language in the case of this paper), and during monitoring prosody.

In the case of video, all the features were below significance level α=0.05 according to the p-value *with* FDR correction. However, the values were relatively close to this threshold. On the other hand, the p-values without FDR correction met the requirements of the *α* below 0.05 (see Table 8, video section).

**Table 8:** Results of the statistical analysis of features and their significance to distinguish between PD patients and HC.

Modality	Features	p-value	p-value_FDR	Median (PD)	Median (HC)	IQR (PD)	IQR (HC)
AUDIO	relF0SD (TSK7)	2.7E-05	0.0057	-0.0408	0.0033	0.0863	0.102
	shimmer (TSK15)	4.6E-05	0.0057	-4.2218	3.374	12.4081	11.1142
	DUV (TSK7)	7.6E-05	0.0062	-4.6691	-1.7888	4.7326	8.6902
	relF0SD (TSK24)	0.000128	0.0078	-0.0389	0.0108	0.1234	0.0828
	shimmer (TSK17)	0.00035	0.0172	-3.7772	3.4596	13.5591	11.1147
	shimmer (TSK13)	0.000581	0.0237	-2.7845	2.5983	11.9312	9.9691
	NSR (TSK25)	0.001787	0.0487	-0.0402	-1.3382	3.7665	2.9786
	DUV (TSK8)	0.001657	0.0487	-4.6784	0.8537	16.464	13.3974
	shimmer (TSK16)	0.001754	0.0487	-2.9298	1.5677	12.2635	16.6985
	NSR (TSK41)	0.002379	0.0571	0.3378	-0.9651	3.1651	2.2758

VIDEO	Features	p-value	p-value_FDR	Median (PD)	Median (HC)	IQR (PD)	IQR (HC)
	rsdD8 (TSK31)	0.000015	0.0733	-2.0961	1.2194	7.9407	7.0653
	rsdEYE18 (TSK31)	0.000015	0.0733	-2.0961	1.2194	7.9407	7.0653
	slopeM7 (TSK41)	0.000021	0.0733	0.0005	-0.0008	0.0024	0.0026
	rsdD8 (TSK32)	0.000025	0.0733	-2.1191	1.4171	6.2564	8.0297
	rsdEYE18 (TSK32)	0.000025	0.0733	-2.1191	1.4171	6.2564	8.0297
	stdD6 (TSK32)	0.000028	0.0733	-0.0111	0.0083	0.0239	0.0337
	aeEYE16 (TSK37)	0.000032	0.0733	-0.0446	0.0449	0.1445	0.1318
	varD6 (TSK32)	0.000034	0.0733	-0.0015	0.0001	0.0014	0.0032
	varM2 (TSK12)	0.000035	0.0733	-0.0013	-0.0003	0.0010	0.0015
	meanM5 (TSK18)	0.000044	0.0733	0.0416	0.1375	0.1433	0.0920

**Table 9:** Accuracy of Parkinson detection from different modalities.

Modality	Accuracy (balanced)	Sensitivity	Specificity	MCC
Speech	0.77 (0.11)	0.81 (0.12)	0.73 (0.19)	0.54 (0.21)
Video	0.81 (0.13)	**0.88 (0.12)**	0.74 (0.23)	0.64 (0.24)
Multimodality	**0.83 (0.11)**	0.88 (0.13)	**0.78 (0.20)**	**0.68 (0.22)**

**Table 10:** Accuracies for the best speech exercises based on video.

Exercise	Accuracy (balanced)	Sensitivity	Specificity	MCC
TSK39	**0.73 (0.14)**	0.78 (0.17)	0.67 (0.24)	**0.47 (0.29)**
TSK41	0.73 (0.13)	0.79 (0.16)	0.66 (0.21)	0.47 (0.26)
TSK40	0.72 (0.15)	0.79 (0.17)	0.65 (0.26)	0.46 (0.30)
TSK4	0.72 (0.13)	**0.81 (0.14)**	0.63 (0.23)	0.46 (0.27)
TSK9	0.72 (0.13)	0.79 (0.15)	0.65 (0.22)	0.45 (0.26)
TSK13	0.71 (0.15)	0.79 (0.17)	0.62 (0.25)	0.43 (0.30)
TSK23	0.71 (0.15)	0.80 (0.14)	0.62 (0.25)	0.44 (0.30)
TSK8	0.71 (0.14)	0.80 (0.15)	0.62 (0.24)	0.44 (0.29)
TSK1	0.71 (0.13)	0.72 (0.18)	**0.69 (0.21)**	0.42 (0.26)
TSK35	0.71 (0.13)	0.78 (0.15)	0.64 (0.22)	0.42 (0.26)

**Table 11:** Accuracies for the best speech exercises based on audio.

Exercise	Accuracy (balanced)	Sensitivity	Specificity	MCC
TSK7	**0.68 (0.13)**	0.71 (0.15)	**0.66 (0.22)**	**0.36 (0.26)**
TSK24	0.67 (0.12)	**0.77 (0.13)**	0.57 (0.22)	0.35 (0.25)
TSK14	0.66 (0.12)	0.70 (0.15)	0.61 (0.20)	0.31 (0.24)
TSK19	0.66 (0.11)	0.67 (0.14)	0.65 (0.21)	0.32 (0.22)
TSK15	0.64 (0.12)	0.75 (0.12)	0.53 (0.23)	0.28 (0.25)
TSK37	0.62 (0.14)	0.64 (0.14)	0.61 (0.21)	0.24 (0.27)
TSK41	0.62 (0.14)	0.65 (0.15)	0.59 (0.23)	0.23 (0.27)
TSK42	0.62 (0.13)	0.73 (0.14)	0.51 (0.22)	0.24 (0.27)
TSK11	0.61 (0.13)	0.64 (0.13)	0.58 (0.21)	0.21 (0.26)
TSK22	0.61 (0.12)	0.66 (0.16)	0.56 (0.21)	0.22 (0.24)

**Table 12:** Accuracies for the best speech exercises based on multimodality.

Exercise	Accuracy (balanced)	Sensitivity	Specificity	MCC
TSK41	**0.74 (0.13)**	0.79 (0.15)	0.68 (0.22)	**0.49 (0.27)**
TSK23	0.73 (0.15)	**0.83 (0.14)**	0.62 (0.26)	0.47 (0.32)
TSK39	0.73 (0.14)	0.78 (0.17)	0.67 (0.24)	0.47 (0.29)
TSK18	0.73 (0.13)	0.78 (0.16)	0.68 (0.23)	0.48 (0.27)
TSK40	0.72 (0.16)	0.80 (0.16)	0.64 (0.25)	0.46 (0.32)
TSK8	0.72 (0.14)	0.81 (0.15)	0.63 (0.24)	0.45 (0.28)
TSK22	0.72 (0.14)	0.75 (0.17)	**0.69 (0.24)**	0.44 (0.28)
TSK4	0.72 (0.13)	0.82 (0.15)	0.62 (0.24)	0.46 (0.27)
TSK9	0.72 (0.13)	0.78 (0.16)	0.65 (0.21)	0.45 (0.25)
TSK1	0.71 (0.13)	0.72 (0.18)	0.69 (0.21)	0.42 (0.26)

**Table 13:**
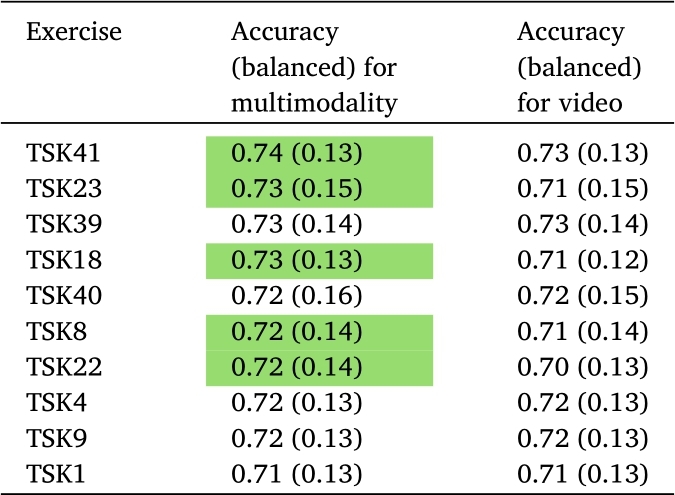
Comparison of the results obtained from multimodal and video approaches.

This criterion was fulfilled by the rsds or uncertainty of information (approximate entropy) in moving eyelids during hard-to-pronounce words or sentences. The second group of features was connected to the movement of the mouth during pronunciation of a tongue twister, difficult words, the vowel ‘u’, and the diadochokinesis exercise and was registered for the slope of the fitted linear regression function and variance parameters. For a feature preselection, the mRMR method was used. We selected between 2 and 5 the most statistically significant audio features from 50 original features for the multimodal approach (48 video, 2 audio).

According to the analysis of the speech exercises, the most valuable features for video modality were found to be mainly tongue twisters, as well as the pronunciation of the vowel ‘e’, the sentence indicated for intonation variability. For the audio models, the best-balanced accuracy was achieved with speech exercises such as vowels, intonation variability tasks, reading poems and tongue twisters. After a combination of audio and video features, the best results were achieved with tongue twisters, diadochokinesis tasks, and the pronunciation of some vowels. To summarize these findings, tongue twister tasks have immense potential for prediction. Especially, difficulty in pronouncing tongue twisters and impairment of the muscles (facial bradykinesia likewise HD) is supposedly an explanation why the tongue twisters could serve as a good clinical tool in prediction PD. It seems that the vowel pronunciation is easier to incorporate into the mHealth system, however, the prediction based on them is less accurate than based on tongue twisters.

To make the model interpretable, SHAP values were used. For the video modality, the limited movement of the jaw in the vertical axis during pronunciation of vowel ‘e’ (varD9 and aeD9 (TSK4), meanD9 (TSK9)) was detected. Additionally, for vowel ‘e’, limited width of opened mouth (minM5 (TSK4)) was observed. Interestingly, a decrease in blinking was characteristic in the pronunciation of difficult sentences and words (aeEYE16 (TSK37), rsdEYE18 (TSK32), maxD5 (TSK31)). However, the higher uncertainty of information for vowels (aeEYE12 (TSK13)) was registered. Moreover, a higher degree of information was registered for the tongue twister, which was fitted by the linear regression function with changes in the oblique distance of the mouth (slopeM7 (TSK41)). Presumably, this could be explained by keeping the mouth open for a longer time without closing and by smaller changes of the mouth in amplitude during a recording of PD patients. For the multimodal model, the values presented by SHAP analysis are covered partially with the outcomes from the video modality. Additionally, for the multimodality, a smaller mouth opening was observed during the pronunciation of the difficult word (maxM3 (TSK31)) or pronunciation of the vowel ‘u’ (varM6 (TSK12)). The audio feature was included, i.e., mean harmonic-to-noise-ratio (mean HNR (TSK15)) during pronunciation of the vowel ‘i’. A similar correlation was observed in [110], [111].

A couple of separate speech exercises (in particular tongue twisters) were recognized to be valuable for video and multimodality. For the video approach, several dependencies were observed, such as smaller amplitude and maximal opening of the mouth in width (rangeM5, maxM5), and more frequently, open eyes (aeREYE_AREA). There was also a lower blinking ratio (maxD6, minEYE1, minEYE22). Additionally, significantly different angles between eyebrows (features aeEYEBROW5, varEYEBROW3) were observed between groups. The best results for the multimodal approach was registered for another tongue twister. It was also observed that PD patients have a lower blinking rate, which was observed using the aeEYE9, aeEYE5, meanEYE5 and slopeEYE18 features. Other significant features are related to the movement of the mouth (slopeM7, slopeD4), where PD patients have been identified as having a smaller range and slower movement as well. The higher the slope, the higher possibility of fluctuations from repeatability from time series. Thereby, there is a higher probability of having PD. Moreover, the differences in the distance between the outer corner of the mouth and eye for the same side (feature meanD2) varied between the groups; and the ratio between those distances on two sides (maxRATIO_FACE). The generated eye-related facial features are similar, however, it was a chance to identify the most valuable in the set of them.

For the audio modality, we merged all the extracted features. We found that relative std of fundamental frequency was negatively correlated with monitoring prosody (relF0SD (TSK24)) and during pronunciation of vowel ‘u’ (relF0SD (TSK7)). The authors of [112] also observed lower values of relF0SD for PD patients; however, this was in connection to patients' tiredness. Moreover, the lower mean value of relF0SD was observed for pronouncing the vowel ‘a’ and reading text among Czech PD patients [113].

In the exercise with the pronunciation of vowels ‘i’, ‘e’, and ‘u’, we identified shimmer (TSK15, TSK17) and jitter (TSK14) to be negatively correlated. Nonetheless, when taking care of gender, the higher values are represented by men patient's than HC likewise lower values have women with PD than HC [114]. One explanation of this could be that the regression out was used when the influence of age and gender was removed from data in our case. With the connection to a difficult-to-pronounce word (TSK30), we observed that the net speech rate (NSR) was found to be positively correlated with PD. In [115], it is claimed that depending on the exercise, the values of NSR for PD vs. HC could be negatively correlated or positively correlated as well. This means that it is recommended to start with unified speech exercises to get their clinical meaning. Finally, the values of relative std of the first formant for checking the intonation pattern were positively correlated with PD (relF1SD (TSK21)). In the literature, this same dependency - the higher value of relF1SD with PD, is detected in monologue and reading tasks [111].

To summarize this section, the introduced methodology focuses on detection PD, suitable for ambient assisted living (AAL) solution, with the usage of telemedicine. It is well inception for further evaluation of symptoms of illnesses by neurologists. This proposed solution could facilitate the life of PD patients, their families, and doctors likewise limiting the burden of the healthcare system. Moreover, several of the most important differences between facial movements were detected: a smaller range of the mouth, different blinking rates and angles between eyebrows, differences in the symmetry of the face, and limited movement of the jaw. These facts are confirmed by statements in the literature on PD [11], [12], [17], [20]. The proposed model has clinical explainability and is also supported by literature [51], [59]. What could be stated about the choice of the metrics is that the most informative among all created features were approximate entropy, variance, slope, and rsd.

When considering the exercise when vowel ‘u’ was pronounced (TSK7), there were registered decreases in the following metrics: value for a fraction of locally unvoiced frames (DUV), relative std of the fundamental frequency (relF0SD), and amplitude perturbation quotient (shimmer). The DUV, as well as shimmer, were observed to have higher values for the PD patients [110] than for HC. Once again, the possible explanation of the reverse phenomenon in our case is the application of the regression out technique. The values of shimmer and jitter are strongly correlated with gender [116], so removing this confounder could have a strong influence on the final distribution of the data. The lower values of relF0SD were also registered in [112]. A positive correlation was found for the mean of harmonic-to-noise-ratio, relative std of the first formant (relF1SD), and mean of harmonic-to-noise ratio (mean HNR). These dependencies were also confirmed in [110], [111].

Nonetheless, this study was conducted using a limited number of patients, including 73 PD subjects and 46 HC subjects. Nevertheless, this dataset is relatively big when compared to the datasets already used for PD detection based on hypomimia symptom (see Table 1). Some parameters like shimmer occur to be lower among PD patients than in HC. It could be caused by the applied regression out method and gender issue [116]. To transfer this solution into clinical practice, the methodology should be trained using an extended dataset. Actigraph, sleep patterns analysis, and brain imaging techniques are still considered to be more accurate. On the other hand, those methods are often expensive and not easily accessible in comparison to the introduced approach based on video and audio automatic analysis. Moreover, some of the participants were wearing glasses during the conduction of the experiment. The achieved results could have been better if the participants had not worn the glasses. Nonetheless, some of the speech exercises required the reading of the prepared text. Nevertheless, the applied approach kept a balance between accuracy and standard conditions. Moreover, the special interest deserves the detection of the progress of the disease [117], not only the detection of PD. Nonetheless, this study considers the classification task with the multimodal approach.

The captivating and promising future direction is extending the dataset and analyses in iRBD cases. The possibility to distinguish the cases based on various modalities, including video recordings was explored in [42]. The patients diagnosed with iRBD are at high risk of developing PD [118]. What's more, facial akinesia belongs to the first symptoms of PD among iRBD patients [119]. Additionally, the extension of the dataset brings up the possibility of increasing the accuracy of predictions.

## 5 Conclusion

In this work, we have proposed several speech exercises and support decision methodologies for PD detection based on computational approaches, which is combining video, audio, and multimodal approaches. We illustrated the state-of-the-art approaches for detection PD based on hypomimia and HD. The collected dataset contains records of 73 PD patients and 46 HC individuals. This unique dataset is relatively large in terms of number of participants as well as number of generated features. In comparison to the dataset presented in the literature, where the authors mostly implemented a single-modal approach with the hypomimia as the only symptom for PD detection, this work brings a novel and more accurate approach (see Table 1). Moreover, this research analyzed 43 speech exercises, what is a significant advantage of this study. Furthermore, we identified as the most accurate approaches the XGBoost models trained on the set of audio and/or video features. We correctly detected PD with 0.83 balanced accuracy, 0.88 sensitivity and 0.78 specificity thanks to the proposed multimodal methodology. The outcome for just the video modality was equal to 0.81 balanced accuracy, whereas for only the audio modality achieved 0.77 balanced accuracy. What is more, we proved that the approaches based on multimodality performed better than those on a single modality. The feature selection step allows us for choosing the best of them and obtaining better models in terms of accuracy. Moreover, the models were additionally trained with features extracted for separate speech exercises. For the step of feature extraction, we tested several combination forms on facial landmarks, statistical measurements, and other metrics. We found that the most valuable features are based on the combination of slope, approximate entropy, and variance. These values were computed for time series containing information on various distances of mouth, eyelid, angles between eyebrows, and metrics linked with facial asymmetry and others. Additionally, we determined which features in the univariate tests show the statistical difference between a group of PD patients and HC. Furthermore, we have indicated what kind of speech exercises would be the most informative and potentially suitable for transferring into a mHealth solution. We identified tongue twisters as the finest for this purpose. The model created for the best tongue twister achieved 0.74 balanced accuracy, 0.79 sensitivity, and 0.68 specificity thanks to the multimodal approach. The models generated for this same speech exercise, but for a single modality achieved 0.73 balanced accuracy for video modality and 0.62 balanced accuracy for audio modality.

The difficulties connected with the pronunciation of the tongue twisters revealed symptoms of hypomimia and HD, which were proved to be valuable in detecting PD. Moreover, we presented the clinical understanding behind the models, which should make the models more valuable in clinical practice. We confirmed the statements about manifesting symptoms of PD existing in literature thanks to the used interpretable models, occurred findings from them, and statistical analysis.

Nonetheless, to transfer this solution into the clinic, the proposed models would have to be trained on a larger dataset. However, this methodology seems to show great promise and deserves further exploration.

## List of acronyms

**3D**: three dimensional
**ACE-R**: Addenbrooke's Cognitive Examination-Revised
**AKV**: absolute kinematic velocity
**AU**: Action Unit
**AAL**: ambient assisted living
**ae**: approximate entropy
**AUC**: Area Under the Curve
**AUROC**: area under the receiver operating characteristic
**BDI**: Beck Depression Inventory
**CNN**: convolutional neural network
**DNN**: deep neural networks
**DX**: Dysarthria Index
**EMG**: electromyography
**ET**: essential tremor
**FACS**: facial action coding system
**fEMG**: facial electromyography
**FECF**: facial expression change factor
**FEF**: facial expression factor
**FER**: facial expression recognition
**FDR**: false discovery rate
**FPS**: frames per second
**FOG**: freezing of gait
**F0**: fundamental frequency
**GNE**: glottal-to-noise excitation
**GRAD-CAM**: Gradient-weighted Class Activation Mapping
**HC**: healthy control
**HBNN-C**: Hierarchical Bayesian neural network
**HOG**: histogram of oriented gradients
**HD**: hypokinetic dysarthria
**iRBD**: idiopathic rapid eye movement sleep behavior disorder
**IQR**: interquartile range
**KNN**: k-nearest neighbors algorithm
**LED**: Levodopa Equivalent Dose
**LID**: levodopa-induced dyskinesia
**ML**: machine learning
**MCC**: Matthews correlation coefficient
**MAX**: maximally discriminative facial movement coding systems
**max**: maximum
**mRMR**: maximum relevance minimum redundancy
**MAE**: Mean Absolute Error
**MFCC**: mel-frequency cepstral coefficients
**MMSE**: Mini-Mental State Examination
**min**: minimum
**mHealth**: mobile health
**MMC**: mobile monitoring and care system
**MDS-UPDRS**: Movement Disorder Society-Sponsored Revision of the Unified Parkinson's Disease Rating Scale
**NMSS**: Non-Motor Symptoms Scale
**NN**: neural network
**PD**: Parkinson's disease
**PCA**: Principal Component Analysis
**PCS**: Progressive Confidence Strategy
**RF**: Random Forest
**REM**: rapid eye movement sleep
**rsd**: relative standard deviation
**RBDSQ**: REM sleep behavior disorder screening questionnaire
**SFHR-NET**: Semantic Feature based Hypomimia Recognition Network
**SF-C**: Semantic Feature Classifier
**se**: Shannon entropy
**SHAP**: SHapley Additive exPlanations
**STFT**: short-time Fourier transform
**std**: standard deviation
**SMOTE**: Synthetic Minority Oversampling Technique
**SVM**: Support Vector Machines
**UPDRS**: Unified Parkinson's Disease Rating Scale
**var**: variance
**VGG**: Visual Geometry Group

## Ethics approval

Data collection was approved by the Masaryk University Ethics Committee under the NT13499 project.

## Code availability

The code is not available.

## CRediT authorship contribution statement

**Justyna Skibińska:** Conceptualization, Data curation, Formal analysis, Funding acquisition, Investigation, Methodology, Project administration, Resources, Software, Validation, Visualization, Writing – original draft, Writing – review & editing. **Jiri Hosek:** Formal analysis, Supervision, Writing – original draft, Writing – review & editing.

## Declaration of Competing Interest

The authors declare that they have no known competing financial interests or personal relationships that could have appeared to influence the work reported in this paper.

## Data Availability

The data are not available. The data that has been used are confidential.

## Footnotes

1 https://www.parktest.net.

2 https://pypi.org/project/face-recognition/.

3 https://translate.google.com/?hl=pl#view=home&op=translate&sl=cs&tl=en.
